# Design and Evaluation of Antimalarial Peptides Derived from Prediction of Short Linear Motifs in Proteins Related to Erythrocyte Invasion

**DOI:** 10.1371/journal.pone.0127383

**Published:** 2015-06-03

**Authors:** Alessandra Bianchin, Angus Bell, Anthony J. Chubb, Nathalie Doolan, Darren Leneghan, Ilias Stavropoulos, Denis C. Shields, Catherine Mooney

**Affiliations:** 1 Conway Institute of Biomolecular and Biomedical Science, University College Dublin, Dublin, Ireland; 2 Complex and Adaptive Systems Laboratory, University College Dublin, Dublin, Ireland; 3 School of Medicine and Medical Science, University College Dublin, Dublin, Ireland; 4 Department of Microbiology, School of Genetics and Microbiology, Moyne Institute of Preventive Medicine, Trinity College, Dublin, Ireland; Oklahoma State University, UNITED STATES

## Abstract

The purpose of this study was to investigate the blood stage of the malaria causing parasite, *Plasmodium falciparum*, to predict potential protein interactions between the parasite merozoite and the host erythrocyte and design peptides that could interrupt these predicted interactions. We screened the *P. falciparum* and human proteomes for computationally predicted short linear motifs (SLiMs) in cytoplasmic portions of transmembrane proteins that could play roles in the invasion of the erythrocyte by the merozoite, an essential step in malarial pathogenesis. We tested thirteen peptides predicted to contain SLiMs, twelve of them palmitoylated to enhance membrane targeting, and found three that blocked parasite growth in culture by inhibiting the initiation of new infections in erythrocytes. Scrambled peptides for two of the most promising peptides suggested that their activity may be reflective of amino acid properties, in particular, positive charge. However, one peptide showed effects which were stronger than those of scrambled peptides. This was derived from human red blood cell glycophorin-B. We concluded that proteome-wide computational screening of the intracellular regions of both host and pathogen adhesion proteins provides potential lead peptides for the development of anti-malarial compounds.

## Introduction

Malaria was the underlying cause of death for 1.24 million people in 2010, the vast majority of these deaths were in Africa and can be attributed to the parasite causing the most virulent form of malaria in humans, *P. falciparum* [1]. *P. falciparum* has an extremely complex life cycle that involves different cells and organ systems in both the female *Anopheles* mosquito and human host. When the mosquito bites the human host, *P. falciparum* sporozoites present in the mosquito salivary glands are passed into the human subcutaneous tissue and are quickly passed into the bloodstream and on to the liver where they invade the hepatocytes. During this stage the sporozoites develop into pre-erythrocytic schizonts that produce new merozoites. The hepatocyte containing the schizont ruptures and the merozoites re-enter the bloodstream where they bind to and invade erythrocytes and also undergo asexual replication, producing more merozoites. At this stage the merozoites can also form gametocytes which, when ingested by another mosquito feeding on the host, will form gametes which will be fertilised into zygotes. The zygotes penetrate the mosquito midgut wall as ookinetes and subsequently oocysts are formed in which replication of new sporozoites occurs. When the oocysts burst the sporozoites are released into the body cavity of the mosquito and invade the mosquito salivary glands where they are ready to restart the cycle. The characteristic symptoms of malaria are caused by the blood stage of the life cycle and it is the point at which the merozoites invades the erythrocytes that is under investigation here.

The merozoite is the first cell of the asexual blood stage of malarial infection and is responsible for establishing new infections in erythrocytes, an essential step in the disease pathogenesis of malaria [2]. The cell contains several characteristic organelles, many of which are in the characteristic apical complex, including rhoptries, micronemes and dense granules. Many of the merozoite proteins that are implicated in the invasion of erythrocytes are located in these apical organelles. Merozoite invasion is a multi-step process that begins with a weak attachment of the merozoite to the erythrocyte surface, reorientation, tight junction formation and subsequent invasion [3]. This whole process is extremely efficient and lasts just a matter of seconds. The surface of the merozoite is covered in membrane proteins such as the glycosylphosphatidylinositol (GPI) anchored merozoite surface proteins (MSP) and erythrocyte binding antigens (EBA). Of these MSP-1 is the most abundant and is thought to play a leading role in the initial contact with the erythrocyte [3]. Many other peripheral proteins such as MSP-7 are thought to associate with MSP-1 [4]. However, there is much difficulty in establishing which merozoite proteins are playing a recognition role and thus contributing to the invasion of erythrocytes and this is largely due to the low affinity nature of the interactions. Although this process is important in targeting erythrocytes that are invasion competent, the next step of invasion could provide a more promising target for intervention. After reorientation of the merozoite, so that the apical prominence is in contact with the erythrocyte membrane, tight junction formation is triggered. The merozoite then secretes its rhoptry contents into the erythrocyte. Many proteins have been predicted to have a role in invasion [5], and some have been shown to be critical for invasion at this stage [6–10].

Peptides have been extensively evaluated as potential antimalarial agents [11] and the idea of blocking erythrocyte invasion using peptides based on key regions of merozoite surface molecules has been explored in dozens of papers. The concentrations of peptide required for inhibiting invasion (or, to be more precise, development of new ring-infected erythrocytes from schizont-infected erythrocytes) in culture have been typically >1 *μ*M and often >100 *μ*M, and in some cases only partial inhibition was achieved. Some published studies lack controls using scrambled peptides of identical amino acid composition to the test peptide, which makes them more difficult to evaluate. A variety of different molecular interactions have been targeted. One of the better characterised examples of merozoite peptides is the 20-mer named R1, based on an erythrocyte binding region of apical membrane antigen 1 (AMA1), that inhibited invasion with an IC_5__0_ of 4 *μ*M [12]. In far fewer cases have peptides based on the known erythrocyte surface receptors for merozoite proteins been investigated: an example is Band 3 [13]. The therapeutic potential of invasion-blocking peptides is still a long way from being realised.

The full extent of the role that these peptides could play in the *P. falciparum* invasion of erythrocytes has yet to be fully examined. There has been significant difficulty in researching the merozoite-erythrocyte interaction in cultured parasites due to the rapid sequence of events from transient initial recognition to tight junction formation, and the difficulty in producing highly purified proteins that will bind with sufficient affinity for analysis. In a proteome of potentially more than 5,000 proteins, this research can be performed more efficiently using bioinformatics tools (i.e. in silico discovery), followed by in vitro testing of a small set of predicted peptides.

Here, predicting regions with SLiM-like properties was used to identify peptides that could be involved potentially in the interaction between the host erythrocyte and the parasite merozoite [14]. SLiMs are short stretches of a protein sequence consisting of about three to ten amino acids [15]. SLiMs have high levels of conservation in comparison to their surrounding residues, they evolve convergently, usually occur in disordered regions [16] and often form a secondary structure when they bind to their interaction partner. There are four classes of SLiMs and they are categorised in relation to their function: ligand sites, which mediate binding of a ligand protein to its corresponding interaction partner, post-translational modification sites, proteolytic cleavage sites and subcellular targeting motifs [17]. Many strategies for targeting adhesion molecules or other receptors involved in pathogenicity focus on compounds that bind to these molecules extracellularly. Here, we instead focused on SLiMs that are involved in the intracellular parts of those transmembrane proteins. For well characterised proteins that have a primary role in signaling, this makes sense, since their intracellular regions are typically engaged in transmitting the signal. However, many adhesion molecules not only adhere, but also transmit a signal or alter the binding of other molecules that contribute to the larger protein complexes that form and facilitate shape change or other cellular change upon binding, as seen in integrin or cadherin proteins. Targeting of receptor molecules by palmitoylated peptides modeled on sections of their intracellular regions has been shown to facilitate alteration of receptor signaling [18], as well as altering responses mediated by proteins which play a role in adhesion [19, 20]. Such peptides can internalize [21], consistent with a model of their location on the intracellular leaflet of the plasma membrane where they perturb in some way the normal signaling [22–24]. Accordingly, we decided to adopt this strategy to determine if peptide targeting of computationally predicted intracellular SLiMs from transmembrane proteins may prove a useful strategy in modulating the merozoite invasion process. The location of the intracellular SLiMs including their distance from a transmembrane (TM) domain and the parent protein membrane topology were taken into account in predicting their suitability as candidates for peptide design. We then palmitoylated the peptides so they could be targeted to the membrane [22, 25] potentially interfering with the binding of the merozoite to the erythrocyte and thus preventing invasion. A small set of the most likely candidates was then tested against cultured *P. falciparum*.

## Materials and Methods

### Peptide Design

We took a three-pronged approach in our search for potential peptides. The first was a whole *P. falciparum* proteome search. The second and third approaches were targeted towards *P. falciparum* proteins and human erythrocytic proteins, which are known to be, or predicted to be, involved in invasion.

We created our first dataset using 1,063 merozoite protein sequences obtained from PlasmoDB [26]. We submitted these sequences to the SCAMPI webserver [27], which predicted TM domains in 357 of these sequences. We focused our search on sequences with a predicted TM domain because SLiMs have been shown to be enriched on the cytoplasmic side of TM proteins [28] and palmitoylated peptides can be targeted to membranes [22, 25]. We then used SLiMPred [14] to predict SLiM like regions in these sequences. We filtered the predictions keeping only those where at least five residues in a row had a predicted probability of being in a SLiM of 0.5 or greater, and these residues fell within a region that was less than 15 residues from a predicted TM domain. In all, 33 predicted SLiMs in 30 sequences remained, which we examined in detail. We chose regions where the motif was predicted to be on the cytoplasmic side of a TM protein, close to the membrane. These peptides were then checked for solubility and synthesisability [29]. Finally five peptides were selected for testing from four protein sequences (see Table 1, Set 1).

**Table 1:** Palmitoylated peptides designed around cytoplasmic SLiMs from transmembrane proteins.

Number	Sequence	Parent Protein
**Set 1:** ***P. falciparum*** **proteins**		
1	Ac-SEQKTPFNINRSK-pal	Putative transporter protein (Figure A in S1 File)
2	pal-KKKLYLYFELFF-NH_2_	Putative protein kinase (Figure B in S1 File)
3	pal-KKKLYLYFE-NH_2_	Putative protein kinase (Figure B in S1 File)
4	pal-KRKLKEEQRTKKIKID	Putative calcium-transporting ATPase (Figure C in S1 File)
5	pal-SSSRKNRFRYLPF-NH_2_	Putative aminophospholipid-transporting P-ATPase (Figure D in S1 File)
5_scr1	pal-FLPRYRFKNSRSS-NH_2_	-
5_scr2	pal-RFNFYSRSRLPKS-NH_2_	-
**Set 2:** ***P. falciparum*** **invasion proteins**		
6	pal-KNSNEPHHIFNIFQK-NH_2_	Reticulocyte binding protein homolog 4 (Figure E in S1 File)
7	pal-KEEIIEIVFDENEEKYF	Reticulocyte binding protein 2 (Figure F in S1 File)
8	Ac-LSESIKNLLKNIYKK-NH_2_	Serine repeat antigen 4 (Figure G in S1 File)
**Set 3: human erythrocyte proteins**		
9	pal-YEKRRKPEDVL-NH_2_	Basigin (Figure H in S1 File)
10	pal-RRLIKKSP-NH_2_	Glycophorin-A (Figure I in S1 File)
10_scr1	pal-RIPRSKLK-NH_2_	-
10_scr2	pal-LKPKIRSR-NH_2_	-
11	pal-SYSIRRLIKA	Glycophorin-B (Figure J in S1 File)
12	pal-SYTIRRLIKA	Glycophorin-B (Figure J in S1 File)
12-Cam	pal-SYTIRRLIKA-NH_2_	Glycophorin-B (Figure J in S1 File)
12_scr1	pal-LKSRIAITYR-NH_2_	-
12_scr2	pal-LITISYKARR-NH_2_	-
12_scr3	pal-IYSLKAIRTR-NH_2_	-
12_scr4	pal-KTSIIALYRR-NH_2_	-
13	pal-KHRKGNNA-NH_2_	Complement receptor type 1 (Figure K in S1 File)

Peptides selected for testing, the peptide sequence showing palmitoylation (pal) acetylation (Ac) and amidation (NH_2_) and the protein from which the peptide originated.

The first peptide, Peptide 1, was selected from a putative 609-residue long *P. falciparum* transporter protein (Q8II64 (PF11_0310)). The SLiM-like region (QKTPF) is found five residues from the TM region predicted by SCAMPI (Figure A in S1 File). We extended the peptide up to the TM region, added a C-terminal lysine and palmitoylation, and a N-terminal acetyl group. Peptides 2 and 3 were selected from the 1,518 residue long putative protein kinase, Q8ILC4 (PF14_0320). We extended the predicted SLiM (YLYFE) to the TM region (adding KKKL), palmitoylated the N-terminus and added a C-terminal amidation—pal-KKKLYLYFELFF-NH_2_ (Figure B in S1 File). As we were concerned that there might be solubility issues with the original peptide we also included a variation of this peptide, which we predicted to be more soluble, since it had three fewer hydrophobic residues (pal-KKKLYLYFE-NH2).

Peptide 4 was selected from the putative calcium-transporting ATPase, Q76NN8. The predicted SLiM (KKIKI) was extended by ten residues to the TM region, and it was palmitoylated at the N-terminus. We did not add a C-terminal amidation because the peptide included the C-terminal residue of the native protein sequence—pal-KRKLKEEQRTKKIKID (Figure C in S1 File). Peptide 5 is taken from the aminophospholipid-transporting P-ATPase, Q8I5L4. The predicted SLiM (RFRYLP) was extended by six residues to the TM region, it was palmitoylated at the N-terminus and amidated at the C-terminus—pal-SSSRKNRFRYLPF-NH_2_ (Figure D in S1 File).

The second set of proteins which we examined were *P. falciparum* proteins that are potentially involved in the invasion of the erythrocyte by *P. falciparum* [3]. Predictions were performed as previously described, without the requirement of being within 15 residues of a predicted TM domain. Three peptides were selected for testing (see Table 1, Set 2). Peptide 6 was selected from reticulocyte binding protein homolog 4 (C0H496/PfRh4). The predicted SLiM (FNIFQ) was extended to the TM region, palmitoylated at the N-terminus and amidated at the C-terminus—pal-KNSNEPHHIFNIFQK-NH_2_ (Figure E in S1 File). Peptide 7 was selected from reticulocyte binding protein 2 homolog (C0H5F4/Rh2b). The predicted SLiM was extended to the C-terminus of the sequence, therefore the peptide was not amidated. However, although we did not extend the peptide to the TM region in this case because the distance was too great and the resulting peptide would have been too long, we did decide to palmitoylate the N-terminus to allow for targeting to the membrane—pal-KEEIIEIVFDENEEKYF (Figure F in S1 File). Peptide 8 was selected from the serine repeat antigen 4 (O96164/SERA-4). The predicted SLiM (LLKNIYKK) was extended seven residues and the peptide was acetylated at the N-terminus and amidated at the C-terminus—Ac-LSESIKNLLKNIYKK-NH_2_ (Figure G in S1 File).

The final set of proteins which we examined were human erythrocytic proteins potentially involved in invasion [3, 30, 31]. The five proteins (basigin, glycophorin-A, -B and -C, and complement receptor type 1) are all TM proteins. We selected peptides from the cytoplasmic side of these TM proteins because SLiMs tend to be enriched in these regions [28]. Preliminary testing of the glycophorin-C peptide (pal-AALQEPALQDAGESSRKEYFI) showed no activity and further tests were not conducted on this peptide. The remaining peptides were as follows (see Table 1, Set 3). Peptide 9 is an 11 residue peptide taken from the C-terminal side of the basigin (P35613) TM protein. We palmitoylated the N-terminus and amidated the C-terminus—pal-YEKRRKPEDVL-NH_2_ (Figure H in S1 File). Peptide 10 is an eight residue peptide taken from the C-terminal side of the glycophorin-A (P02724) TM protein. We palmitoylated the N-terminus and amidated the C-terminus—pal-RRLIKKSP-NH_2_ (Figure I in S1 File). Peptide 11 is taken from the C-terminal side of the glycophorin-B (P06028) TM protein—pal-SYSIRRLIKA. Peptide 12 is a common human polymorphism [32] of peptide 11 where the serine in position three in the peptide has been replaced by threonine—pal-SYTIRRLIKA. Both peptides were palmitoylated but neither was amidated because the peptide extended to the C-terminus of the protein sequence (Figure J in S1 File). The final peptide that we selected for testing, Peptide 13, was from complement receptor type 1 (P17927). The peptide, pal-KHRKGNNA-NH_2_, extends eight residues from the C-terminal side of the TM protein and was palmitoylated at the N-terminus and amidated at the C-terminus (Figure K in S1 File).

### Parasite culture

*P. falciparum* line 3D7, adapted to grow in Albumax® (BioSciences, Dun Laoghaire, Ireland), was cultured in human erythrocytes (Irish Blood Transfusion Board, Ireland) in standard growth medium, in candle jars [33]. Fresh erythrocytes were obtained every three weeks and washed according to lab protocols. Staining of blood smears was with Giemsa stain. The number of infected erythrocytes was counted by microscopy and the parasitaemia was calculated.

### Peptide inhibition of parasite growth

The antimalarial activity of test peptides (ChinaPeptides Co. Ltd, Shanghai, China) was determined in culture on the 3D7 line of *P. falciparum* using the parasite lactate dehydrogenase (pLDH) method in 96-well microtitre plates [34, 35] with modifications as previously described [36]. Each test peptide was initially evaluated at three different concentrations, 100 *μ*M, 10 *μ*M and 1 *μ*M, to determine activity range. The effect of the compounds was evaluated at 48 and 72 hours on growth of asynchronous cultures (cultured as described in the previous section) of *P. falciparum*, determined by the assay of pLDH activity. We used unsynchronised cultures in order (i) to make no prior assumptions about the stage of growth that might be inhibited and (ii) to avoid possible inhibitory effects of the synchronising protocol, e.g. sorbitol treatment, separation techniques, etc. The appropriate dilutions of the compounds were prepared in DMSO and added to the cultures of *P. falciparum* (2% haematocrit, 0.8% initial parasitaemia and a maximum of 0.5% DMSO). The same methodology was then used to assess the dose-response curves of peptides 5, 7, 10, 11, 12, 13 using ten concentrations between 10 *μ*M and 100 *μ*M.

### Synchronisation of cultures

Routine erythrocytic *P. falciparum* cultures are an asynchronous culture consisting of rings, trophozoites, schizonts and merozoites. To enrich for the late stages of erythrocytic development, parasites at high parasitaemia (>15%) were subjected to magnetic selection [37]. Parasitised erythrocytes were passed over a CS column (Miltenyi Biotec, Surrey, UK) which was mounted on a VarioMacs magnet (Miltenyi Biotec, Surrey, UK). The column was then washed with incomplete medium (RPMI 1640 supplemented with 25 mM HEPES, hypoxanthine [50 *μ*g/ml], 0.16% w/v glucose) to remove unparasitised erythrocytes and was then removed from the magnet, a further wash with incomplete medium eluting late-stage parasitised erythrocytes.

### Schizont to ring development assay

Parasites synchronised to late stages (trophozoite/schizont) by magnetic selection were incubated with either the peptide being tested (100 *μ*M concentration) or vehicle only control (DMSO) in complete medium for two hours. Treated schizonts were washed with complete medium and centrifugation at 350 xg for 10 minutes. Washing was repeated twice. Treated schizonts were then introduced to fresh erythrocytes at 2.5% hematocrit and incubated overnight (14–16 hours) with shaking. Parasitaemia was assessed by counting the number of ring stages in parasitised erythrocytes in Giemsa stained blood smears by light microscopy [37]. This crude assay measures not just invasion but merozoite release and early ring-stage development.

### Hemolysis assay

The hemolytic activity of the peptides was investigated using erythrocytes from healthy human volunteers. Blood was collected in the presence of 15% anticoagulant acid citrate glucose (ACD) (38 mM citric acid, 75 mM sodium citrate, 124 mM glucose) and was centrifuged at 150 xg for 10 minutes at room temperature. Plasma was removed and erythrocytes were washed twice with buffer A (pH 7.4) (130 mM sodium chloride, 10 mM sodium citrate, 9 mM sodium biocarbonate, 6 mM glucose, 0.9 mM magnesium chloride, 0.81 mM potassium hydrogen phosphate, 10 mM Tris), by cenrifugation at 1,000 xg for 5 minutes and resuspended in the same buffer. For the hemolysis assay approximately 1.5 × 10^8^ erythrocytes were incubated with solutions of peptides 5, 10 or 12-Cam at 100 *μ*M and 10 *μ*M concentrations for 15 minutes at 37°C. After incubation, samples were centrifuged at 1,000 xg for 5 minutes and the supernatant was analysed for the presence of hemoglobin at an absorbance of 405 nm. Complete hemolysis was achieved using 0.1% (v/v) Triton X-100 yielding the 100% control value. The assay was run in duplicate with three different donors.

## Results

### Peptide inhibition of parasite growth rate

**Fig 1:**
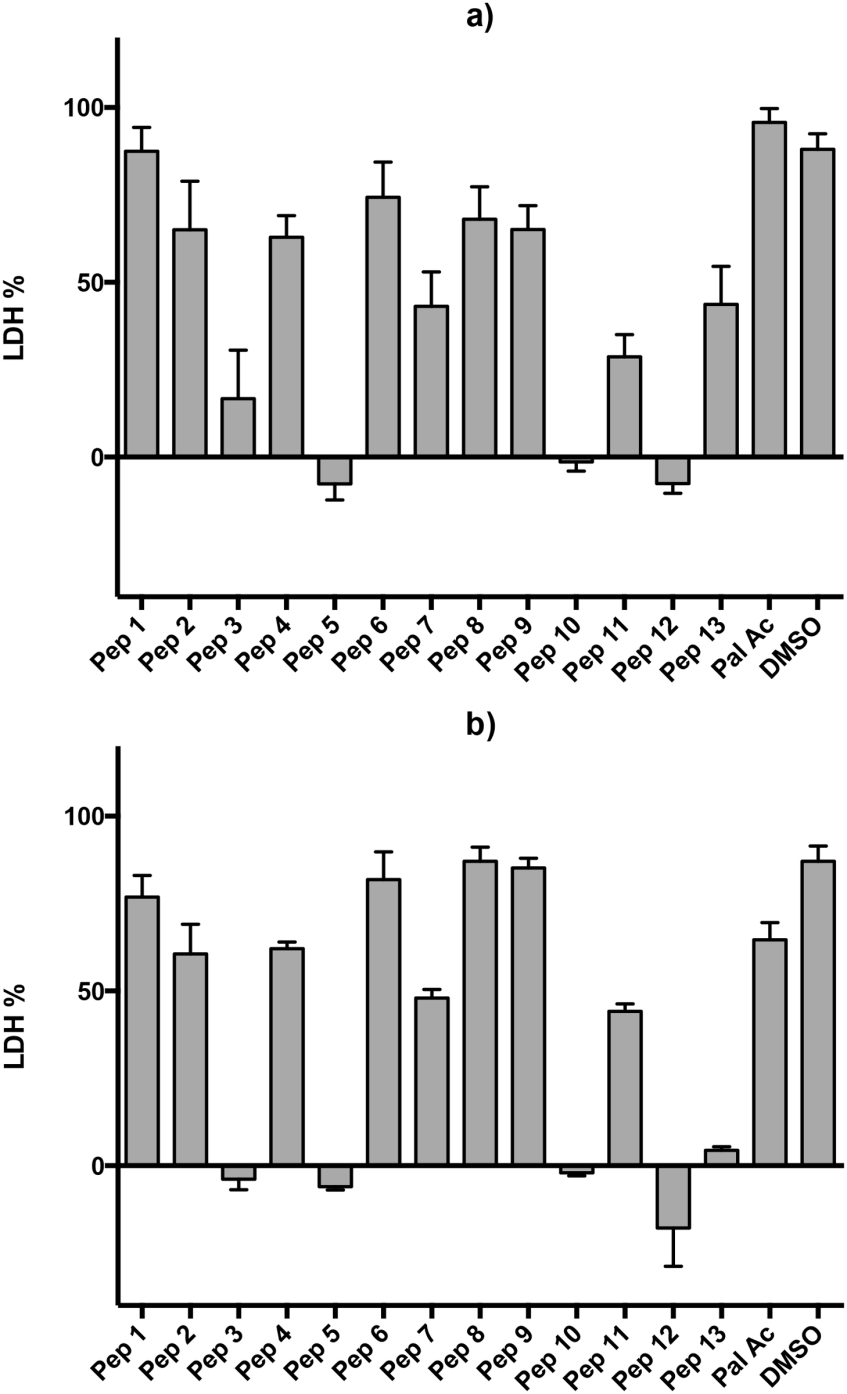
Growth inhibition assay. Peptides were tested at 100 *μ*M. Data were collected at 48 hours (a) and at 72 hours (b). Data are means of three experiments in duplicate and vertical bars indicate standard errors.

We tested the antimalarial activity of the 13 peptides at three different concentrations (Fig 1). As we used asynchronous culture for the growth inhibition assays the experiments were carried out at two time points to ensure that the peptides were tested over the whole parasite life cycle. Growth inhibition was clearly observed at the 100 *μ*M concentration for peptides 5, 10 and 12 at both the 48 hour and 72 hour time points (Fig 1). Peptides 3, 7, 11 and particularly 13 also showed some growth inhibition. These peptides were selected for further investigation (except peptide 3 due to insolubility). At the lowest concentration of 1 *μ*M the activity was absent. Palmitate and DMSO were used as controls and showed no inhibitory effect.

### Dose-response curve confirms inhibitory effects

**Fig 2:**
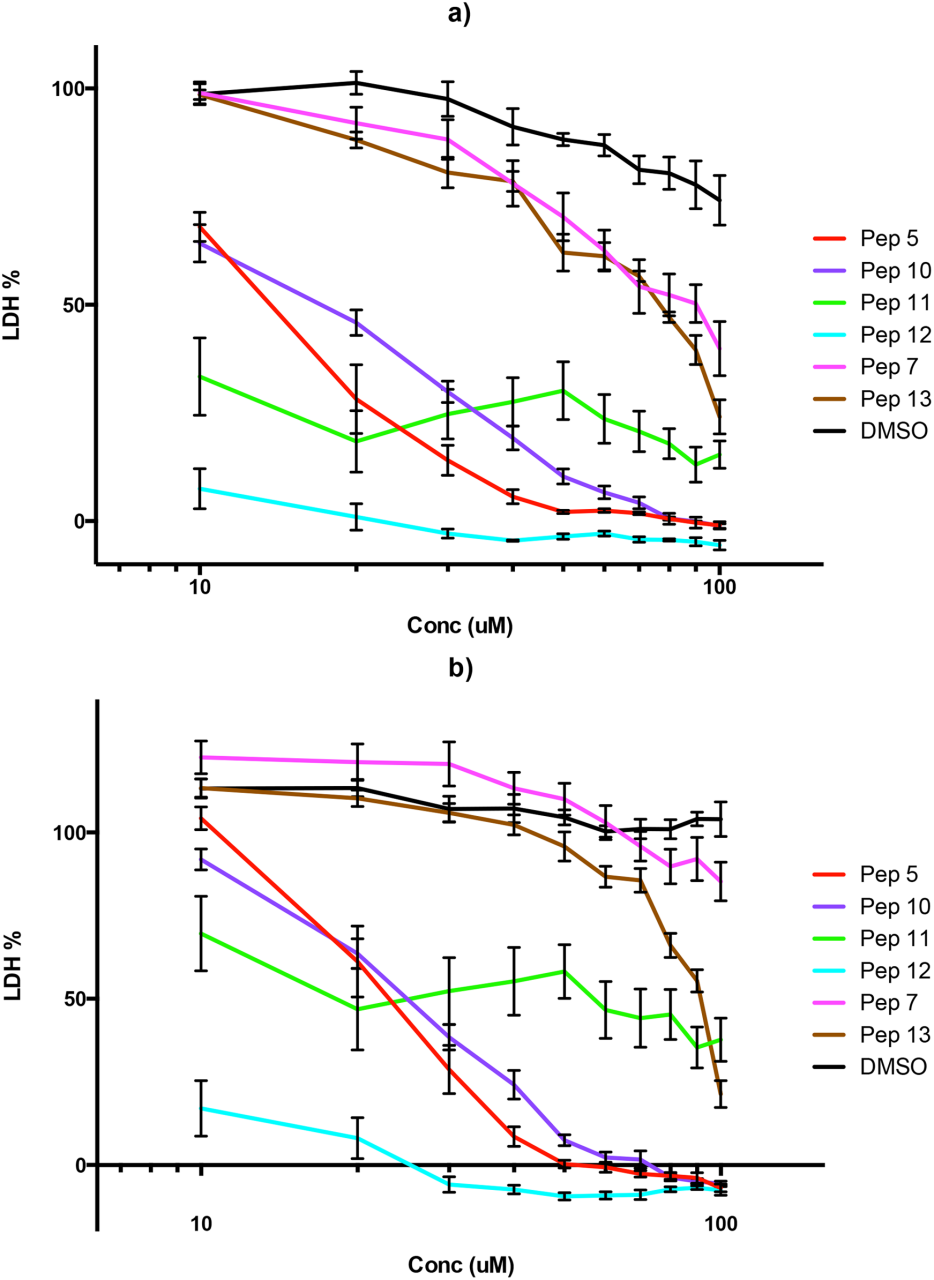
Dose-response curves for selected peptides. Peptides 5, 7, 10, 11, 12, 13 and DMSO were evaluated at 48 hours (a) and at 72 hours (b). Data are means of three experiments in duplicate and vertical bars indicate standard errors.

Because the peptides’ inhibitory activity dropped dramatically between 10 *μ*M and 1 *μ*M, we performed another set of experiments to determine the dose-response curve with 10 data points (Fig 2). Six peptides were tested, the three most active ones (peptides 5, 10 and 12) and peptides 7, 11 and 13. Peptides 7 and 13 were tested because they showed moderate activity at 100 *μ*M, however, the curves confirmed no inhibition at 10 *μ*M. Peptide 11 was tested as a control for peptide 12. The concentration-response curves for peptides 5, 10 and 12 showed clear increases in growth inhibition with increasing concentration, while peptide 11 showed no such clear effect, with a non-monotonic dose response relationship (Fig 2).

An important observation was the discrepancy in activity at 10 *μ*M between the first and second sets of experiments (S1A and S1B Fig, respectively). Peptides 11 and 12 were selected from glycophorin-B (P06028) which is a natural variant in humans for the amino acid in position 84, from serine to threonine. The natural variation is due to a single nucleotide variation C/G. The Population genetics of 1000 Genomes allele frequencies (http://www.1000genomes.org/) show that G is the minor allele with 16% and C is the major allele with 84% frequency; based on that we should expect more people expressing the natural variant T84 corresponding to the sequence of the active peptide 12. It is possible that the donor blood used for the different sets of experiments may be expressing different versions of glycophorin-B, therefore affecting the behaviour of peptide 12, but this requires further investigation.

### Peptide inhibition of parasite “invasion”

**Fig 3:**
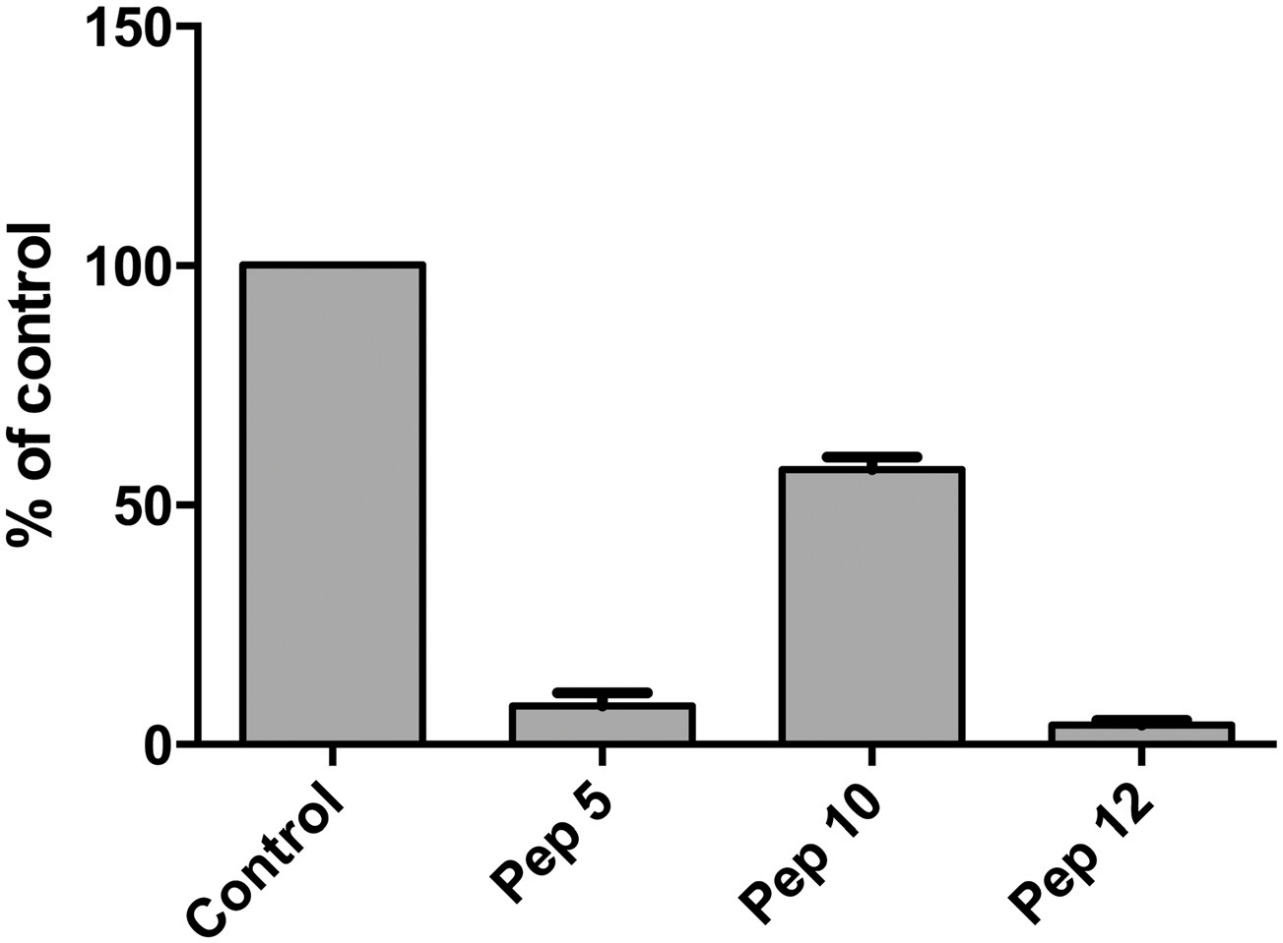
Effect of peptides on merozoite invasion. Vehicle only control (DMSO) and peptides 5, 10 and 12 (100 *μ*M concentration) were incubated for 2 hours with schizonts purified by magnetic selection which were subsequently introduced to fresh erythrocytes. The parasitaemia 14–16 hours later was assessed by counting rings in Giemsa stained blood smears microscopically and expressed as a percentage of the control culture (shown as % of control, y axis). Data are means of two experiments in triplicate and vertical bars indicate standard errors.

We investigated if the peptide growth inhibition could be attributed to inhibition of invasion, or events immediately before or after invasion, by using synchronised parasites cultures and counting the number of freshly infected erythrocytes 14–16 hours after treating late stage parasites with the peptides. Both peptides 5 and 12 almost totally reduced the ability of merozoites to establish new infections in fresh erythrocytes (Fig 3). Peptide 10 also had an effect on the number of new infections but it was not as significant as that of the other two peptides. In the two cultures most affected by the peptides the new ring stage parasites that were counted appeared very unhealthy morphologically (data not shown) and for all intents and purposes the parasitaemia in these cultures was zero. This leads us to the conclusion that peptides 5 and 12 are potent inhibitors of the ability of the parasite to establish new infections in fresh erythrocytes. It should be borne in mind that this assay does not use isolated merozoites and therefore measures late merozoite development and release as well as invasion. Although it is possible that the effects of the peptides occur just before invasion, during invasion and/or after invasion as the parasites develop into visibly normal ring forms this is the method of choice in many publications to assess invasion [38]. A detailed state-of-the-art invasion assay (for example using flow cytometry) would be appropriate for any highly active peptide leads but was beyond the scope of the present study.

### Testing specificity of peptide sequence versus composition

Scrambled peptides were designed for the three most active peptides (5, 10 and 12) (Table 1), to check if the peptide activity was a function of amino acid composition or was sequence specific. For peptide 5 and 10 we selected two scrambled peptides each, with both palmitoylation and amidation; for peptide 12 we selected four scrambled peptides all palmitoylated and amidated. We tested peptide 12 with both palmitoylation and amidation to investigate better the effect of peptide charge.

At 100 *μ*M, all of the scrambled peptides tested had some effect on growth inhibition. This indicates that there is some non-specific contribution to growth inhibition from peptide composition. This appears to relate to the positive charge of all three candidate peptides (5, 10 and 12) which were each very positively charged (net charge of 4, 4 and 3 respectively) and included no negatively charged residues. Previous work has suggested that peptides of low or high net charge are enriched for bioactivity [39]. Some of the inactive peptides tested above also had a number of positively charged residues, which is a common feature of membrane proximal intracellular regions of transmembrane proteins, but these inactive peptides each had a mixture of positive and negative charge. While the inactive peptide 4 had a net positive charge of 5, it had a total of 8 positive and 3 negative charges.

**Fig 4:**
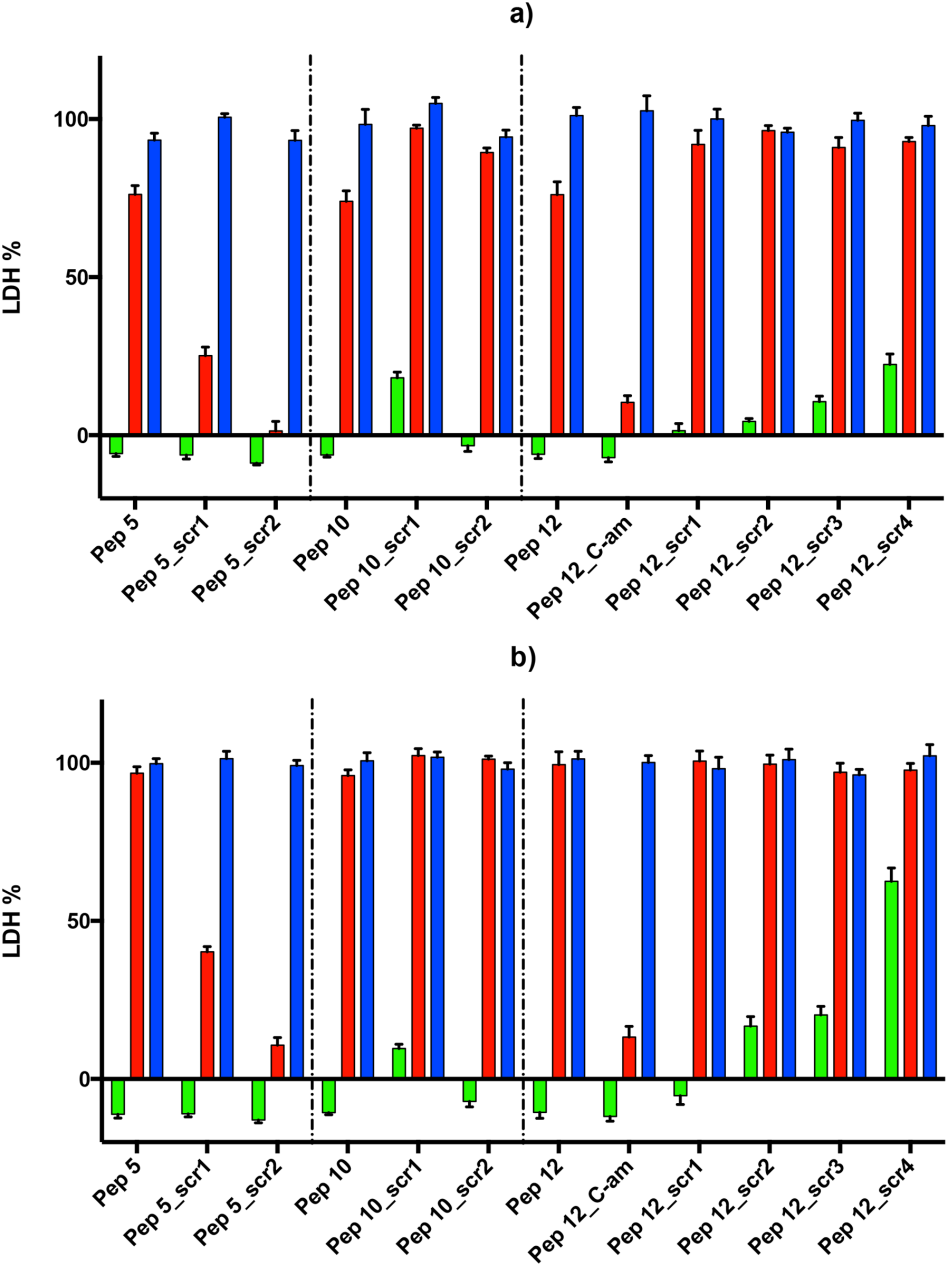
Growth inhibition by scrambled peptides. Peptides 5, 10 and 12 and their scrambled peptides were tested in three concentrations, 100 *μ*M (left bar), 10 *μ*M (middle bar) and 1 *μ*M (right bar) at 48 hours (a) and at 72 hours (b). Data are means of three experiments in duplicate and vertical bars indicate standard errors.

Overall, the results for peptide 5 and 10 were not promising, as the scrambled peptides showed similar or better growth inhibition effect, therefore the effect may be non-specific. Thus, while it is of course possible that these two peptides are indeed acting by mimicking in some way the sequence region of the protein from which they are derived, the fact that scrambled peptides have a similar effect greatly increases the likelihood that the peptides may be acting via some non-specific mechanism, since the number of protein interaction surfaces that involve peptides with a similar amino acid composition greatly exceeds the number of surfaces that share a strongly similar sequence. Interestingly peptide 12 showed more inhibition when amidated and none of the scrambled versions of peptide 12 showed the same degree of inhibition at 10 *μ*M (Fig 4). This is suggestive that at the lower dose non-specific effects are lessened and the sequence-specific effect of peptide 12 is seen.

### Testing for erythrocyte lysis in the presence of peptides

Although the antimalarial effects of the peptides were not considered to be due to erythrocyte lysis, as judged by visual inspection of the assay plates, we performed a hemolysis assay to confirm this. At a concentration of 100 *μ*M peptide 10 caused approximately 50% release of haemoglobin from erythrocytes (compared with 0.1% (v/v) Triton-X100), whereas the release caused by peptides 5 and 12-Cam was between 10% and 20%. All peptides were non-haemolytic at 10 *μ*M concentration (S2 Fig).

## Discussion

We selected 13 computationally predicted peptides from three sources: (i) the whole *P. falciparum* proteome, (ii) *P. falciparum* proteins potentially involved in erythrocyte invasion and (iii) human erythrocytic proteins potentially involved in invasion. Of the 13 peptides tested, three peptides (5, 10 and 12) inhibited parasite growth at the 100 *μ*M concentration at both the 48 hour and 72 hour time points and showed a clear increase in growth inhibition with increasing dose between 10 *μ*M and 100 *μ*M. Peptide 5, derived from the aminophospholipid-transporting P-ATPase protein, was predicted using the whole *P. falciparum* proteome search strategy. This protein is expressed in the intraerythrocytic asexual cycle, and stage-specifically in mature trophozoites and schizonts, with no apparent expression in the ring stage [40]. Peptides 10 and 12 were selected from the human erythrocytic proteins glycophorin A and B respectively.

We then investigated if the peptide growth inhibition could be attributed to inhibition of invasion and demonstrated that peptides 5 and 12 almost totally reduced the ability of merozoites to establish new infections in fresh erythrocytes. However, we were not able to distinguish between a number of events during the late erythrocyte stages up to and immediately after invasion. Therefore, the peptides could be active against the release of mature merozoites by bursting schizonts, the free merozoites themselves, invasion and/or early ring development. It is also possible that the inhibition of parasite growth is not due to inhibition of invasion but due to some other unknown mechanism operating around the same stage. We checked whether the peptide action was simply to lyse the erythrocytes and concluded that the antimalarial effects of the peptides are not primarily due to erythrocyte lysis for peptides 5 and 12-Cam, although it is likely that at 100 *μ*M some of the inhibition of parasite growth rate by peptide 10 is due to hemolysis.

Finally, we tested the antimalarial activity of scrambled peptide versions of peptides 5, 10 and 12. The scrambled peptides 5 and 10 showed similar or better growth inhibition effect as the original peptides, therefore the effect seems to be non-specific and may be related to the positive charge of the peptides. The most promising of the peptides tested was the amidated version of peptide 12, which showed additional activity based on its sequence (i.e. it was more active than four scrambled controls of identical amino acid composition). This suggests that it may indeed be acting by mimicking the sequence of glycophorin B’s short cytoplasmic tail. For these reasons, the major conclusion of our analyses is to strongly support further investigation of peptide 12.

It is intriguing that we see some differences in activity for the two polymorphic versions of the glycophorin-derived peptides 11 and 12. While the amino acid properties are highly similar for the variation (between serine and threonine) we would not anticipate that such a difference might impose a functional effect. Nevertheless, the difference in molecular mass and presentation of these two groups may be having some effect. Targeting pathogen proteins is attractive because it is possible to damage the pathogen without harming host molecular components. However, the ability of pathogens to evolve to avoid strategies designed to limit them can sometimes exceed the ability of the human hosts to develop useful solutions either over evolution or in the clinic. Thus, many genetic variants that alter malarial resistance are in fundamental host cell factors (glucose 6-phosphate dehydrogenase and sickle cell) rather than in components of the immune response mechanisms. It is possible that this particular position may be under some selective adaptive constraint, since the residue is a glycine in chimpanzee, but either serine or threonine in man. However, previous workers have speculated that this variant is not important for malarial resistance, focusing instead on studying extracellular amino-acid altering polymorphisms [41]. It will be of interest to test whether the intracellular Ser/Thr polymorphism encoded by SNP rs1132783 does indeed associate with malarial resistance. If it does, it may well be of strong interest to develop more potent compounds targeting glycophorin B, for which the peptides developed here may act as initial lead compounds.

We conclude that screening for intracellular short linear protein motifs, and then testing their function when delivered into cells as peptides using palmitylation or other cell penetrating strategies, represents an alternative pathogen targeting strategy. This contrasts with the more usual emphasis on targeting the extracellular regions of both host and pathogen receptors. Such screens need to pay careful attention to both sequence-specific and composition-specific effects of peptides that modulate pathogen invasion.

## Supporting Information

S1 File Images from the SLiMPred server showing the predicted SLiMs and peptides. Peptide 1, Ac-SEQKTPFNINRSK-pal Putative transporter protein Q8II64 (PF11_0310) 609 residues in length. Predicted SLiM: QKTPF (residues 78–82) **(Figure A)**. Peptide 2, pal-KKKLYLYFELFF-NH_2_, and Peptide 3, pal-KKKLYLYFE-NH_2_ Putative protein kinase Q8ILC4 (PF14_0320) 1518 residues in length. Predicted SLiM: YLYFE (residues 444–448) **(Figure B)**. Peptide 4, pal-KRKLKEEQRTKKIKID Putative Calcium-transporting ATPase Q76NN8 (PFA0310c) 1228 residues in length. Predicted SLiM: KKIKI (residues 1223–1227) **(Figure C)**. Peptide 5, pal-SSSRKNRFRYLPF-NH_2_ Putative aminophospholipid-transporting P-ATPase Q8I5L4 (PFL0950c) 1555 residues in length. Predicted SLiM: RFRYLP (residues 463–468) **(Figure D)**. Peptide 6, pal-KNSNEPHHIFNIFQK-NH_2_ Reticulocyte binding protein homolog 4 C0H496. 1716 residues in length. Predicted SLiM: FNIFQ (residues 1659–1663) **(Figure E)**. Peptide 7, pal-KEEIIEIVFDENEEKYF Reticulocyte binding protein 2 C0H5F4 (RBP2B_PLAF7) 3179 residues in length. Predicted SLiM: IEIVFDE (residues 3167–3173) **(Figure F)**. Peptide 8, Ac-LSESIKNLLKNIYKK-NH_2_ Serine repeat antigen 4 O96164 (O96164_PLAF7) 962 residues in length. Predicted SLiM: LLKNIYKK (residues 328–335) **(Figure G)**. Peptide 9, pal-YEKRRKPEDVL-NH_2_ Basigin P35613 (BASI_HUMAN) 385 residues in length **(Figure H)**. Peptide 10, pal-RRLIKKSP-NH_2_ Glycophorin-A P02724 (GLPA_HUMAN) 150 residues in length **(Figure I)**. Peptide 11, pal-SYSIRRLIKA and peptide 12, pal-SYTIRRLIKA Glycophorin-B P06028 (GLPB_HUMAN) 91 residues in length **(Figure J)**. Peptide 13, pal-KHRKGNNA-NH_2_ Complement receptor type 1 P17927 (CR1_HUMAN) 2039 residues in length **(Figure K)**.
(PDF)

S1 Fig Concentration-dependent growth inhibition of peptide 12 compared to peptide 11. The graphs present peptides 11 (green) and 12 (blue). a) Growth inhibition assay. Light green and light blue refer to peptide 11 and 12 respectively at 48 hours; dark colours refer to 72 hours of inhibition. b) Dose-response curve with fit line.
(EPS)

S2 Fig Percentage of haemoglobin released from erythrocytes. Peptides 5, 10 and 12-Cam were tested for hemolytic activity. Light grey and red bars represent haemoglobin release when peptides were used at 100*μ*M and 10*μ*M, respectively. Complete hemolysis was achieved using 0.1% (v/v) Triton X-100 yielding the 100% control value. The assay was run in duplicate with three different donors.
(EPS)

## References

[1] Murray CJ, Rosenfeld LC, Lim SS, Andrews KG, Foreman KJ, Haring D, et al Global malaria mortality between 1980 and 2010: a systematic analysis. The Lancet. 2012;379(9814):413–431. 10.1016/S0140-6736(12)60034-8 22305225

[2] Cowman AF, Berry D, Baum J. The cellular and molecular basis for malaria parasite invasion of the human red blood cell. The Journal of Cell Biology. 2012;198(6):961–971. 10.1083/jcb.201206112 22986493 PMC3444787

[3] Cowman AF, Crabb BS. Invasion of red blood cells by malaria parasites. Cell. 2006;124(4):755–766. 10.1016/j.cell.2006.02.006 16497586

[4] Harvey KL, Gilson PR, Crabb BS. A model for the progression of receptor–ligand interactions during erythrocyte invasion by *Plasmodium falciparum* . International Journal for Parasitology. 2012;42(6):567–573. 10.1016/j.ijpara.2012.02.011 22710063

[5] Hester J, Chan ER, Menard D, Mercereau-Puijalon O, Barnwell J, Zimmerman PA, et al De novo assembly of a field isolate genome reveals novel *Plasmodium vivax* erythrocyte invasion genes. PLoS Neglected Tropical Diseases. 2013 12;7(12):e2569 10.1371/journal.pntd.0002569 24340114 PMC3854868

[6] Mitchell G, Thomas A, Margos G, Dluzewski A, Bannister L. Apical membrane antigen 1, a major malaria vaccine candidate, mediates the close attachment of invasive merozoites to host red blood cells. Infection and Immunity. 2004;72(1):154–158. 10.1128/IAI.72.1.154-158.2004 14688092 PMC343990

[7] Baum J, Richard D, Healer J, Rug M, Krnajski Z, Gilberger TW, et al A conserved molecular motor drives cell invasion and gliding motility across malaria life cycle stages and other apicomplexan parasites. Journal of Biological Chemistry. 2006;281(8):5197–5208. 10.1074/jbc.M509807200 16321976

[8] Lamarque M, Besteiro S, Papoin J, Roques M, Vulliez-Le Normand B, Morlon-Guyot J, et al The RON2-AMA1 interaction is a critical step in moving junction-dependent invasion by apicomplexan parasites. PLoS Pathogens. 2011;7(2):e1001276 10.1371/journal.ppat.1001276 21347343 PMC3037350

[9] Besteiro S, Dubremetz JF, Lebrun M. The moving junction of apicomplexan parasites: a key structure for invasion. Cellular Microbiology. 2011;13(6):797–805. 10.1111/j.1462-5822.2011.01597.x 21535344

[10] Srinivasan P, Beatty WL, Diouf A, Herrera R, Ambroggio X, Moch JK, et al Binding of *Plasmodium* merozoite proteins RON2 and AMA1 triggers commitment to invasion. Proceedings of the National Academy of Sciences. 2011;108(32):13275–13280. 10.1073/pnas.1110303108 PMC3156155 21788485

[11] Bell A. Antimalarial peptides: the long and the short of it. Current Pharmaceutical Design. 2011;17(25):2719–2731. 10.2174/138161211797416057 21728986

[12] Harris KS, Casey JL, Coley AM, Masciantonio R, Sabo JK, Keizer DW, et al Binding hot spot for invasion inhibitory molecules on *Plasmodium falciparum* apical membrane antigen 1. Infection and Immunity. 2005 10;73(10):6981–6989. 10.1128/IAI.73.10.6981-6989.2005 16177378 PMC1230972

[13] Goel VK, Li X, Chen H, Liu SC, Chishti AH, Oh SS. Band 3 is a host receptor binding merozoite surface protein 1 during the *Plasmodium falciparum* invasion of erythrocytes. Proceedings of the National Academy of Sciences. 2003 4;100(9):5164–5169. 10.1073/pnas.0834959100 PMC154316 12692305

[14] Mooney C, Pollastri G, Shields DC, Haslam NJ. Prediction of short linear protein binding regions. Journal of Molecular Biology. 2012;415(1):193–204. 10.1016/j.jmb.2011.10.025 22079048

[15] Davey NE, Van Roey K, Weatheritt RJ, Toedt G, Uyar B, Altenberg B, et al Attributes of short linear motifs. Molecular BioSystems. 2012;8(1):268–281. 10.1039/C1MB05231D 21909575

[16] Fuxreiter M, Tompa P, Simon I. Local structural disorder imparts plasticity on linear motifs. Bioinformatics. 2007;23(8):950–956. 10.1093/bioinformatics/btm035 17387114

[17] Dinkel H, Van Roey K, Michael S, Davey NE, Weatheritt RJ, Born D, et al The eukaryotic linear motif resource ELM: 10 years and counting. Nucleic Acids Research. 2014;42(D1):D259–D266. 10.1093/nar/gkt1047 24214962 PMC3964949

[18] O'Callaghan K, Kuliopulos A, Covic L. Turning receptors on and off with intracellular pepducins: new insights into G-protein-coupled receptor drug development. Journal of Biological Chemistry. 2012;287(16):12787–12796. 10.1074/jbc.R112.355461 22374997 PMC3339939

[19] Stephens G, O'Luanaigh N, Reilly D, Harriott P, Walker B, Fitzgerald D, et al A sequence within the cytoplasmic tail of GpIIb independently activates platelet aggregation and thromboxane synthesis. Journal of Biological Chemistry. 1998;273(32):20317–20322. 10.1074/jbc.273.32.20317 9685382

[20] Stavropoulos I, Golla K, Moran N, Martin F, Shields DC. Cadherin juxtamembrane region derived peptides inhibit TGF *β*1 induced gene expression. BioArchitecture. 2014;4(3):103–110. 10.4161/bioa.32143 25108297 PMC4201599

[21] Johannessen L, Remsberg J, Gaponenko V, Adams KM, Barchi JJ, Tarasov SG, et al Peptide Structure Stabilization by Membrane Anchoring and its General Applicability to the Development of Potent Cell-Permeable Inhibitors. ChemBioChem. 2011;12(6):914–921. 10.1002/cbic.201000563 21365731 PMC3493468

[22] Covic L, Misra M, Badar J, Singh C, Kuliopulos A. Pepducin-based intervention of thrombin-receptor signaling and systemic platelet activation. Nature Medicine. 2002;8(10):1161–1165. 10.1038/nm760 12357249

[23] Janz JM, Ren Y, Looby R, Kazmi MA, Sachdev P, Grunbeck A, et al Direct interaction between an allosteric agonist pepducin and the chemokine receptor CXCR4. Journal of the American Chemical Society. 2011;133(40):15878–15881. 10.1021/ja206661w 21905700

[24] Shields DC, O'Brien KT. Pepducins In: AccessScience. McGraw-Hill Education; 2014 p. 914–921.

[25] Edwards RJ, Moran N, Devocelle M, Kiernan A, Meade G, Signac W, et al Bioinformatic discovery of novel bioactive peptides. Nature Chemical Biology. 2007;3(2):108–112. 10.1038/nchembio854 17220901

[26] Aurrecoechea C, Brestelli J, Brunk BP, Dommer J, Fischer S, Gajria B, et al PlasmoDB: a functional genomic database for malaria parasites. Nucleic Acids Research. 2009;37(suppl 1):D539–D543. 10.1093/nar/gkn814 18957442 PMC2686598

[27] Bernsel A, Viklund H, Falk J, Lindahl E, von Heijne G, Elofsson A. Prediction of membrane-protein topology from first principles. Proceedings of the National Academy of Sciences. 2008;105(20):7177–7181. 10.1073/pnas.0711151105 PMC2438223 18477697

[28] Stavropoulos I, Khaldi N, Davey NE, O'Brien K, Martin F, Shields DC. Protein disorder and short conserved motifs in disordered regions are enriched near the cytoplasmic side of single-pass transmembrane proteins. PLoS One. 2012;7(9):e44389 10.1371/journal.pone.0044389 22962613 PMC3433447

[29] Duffy FJ, Verniere M, Devocelle M, Bernard E, Shields DC, Chubb AJ. CycloPs: generating virtual libraries of cyclized and constrained peptides including nonnatural amino acids. Journal of Chemical Information and Modeling. 2011;51(4):829–836. 10.1021/ci100431r 21434641

[30] Tham WH, Wilson DW, Lopaticki S, Schmidt CQ, Tetteh-Quarcoo PB, Barlow PN, et al Complement receptor 1 is the host erythrocyte receptor for *Plasmodium falciparum* PfRh4 invasion ligand. Proceedings of the National Academy of Sciences. 2010;107(40):17327–17332. 10.1073/pnas.1008151107 PMC2951459 20855594

[31] Crosnier C, Bustamante LY, Bartholdson SJ, Bei AK, Theron M, Uchikawa M, et al Basigin is a receptor essential for erythrocyte invasion by *Plasmodium falciparum* . Nature. 2011;480(7378):534–537. 10.1038/nature10606 22080952 PMC3245779

[32] Kudo S, Fukuda M. Structural organization of glycophorin A and B genes: glycophorin B gene evolved by homologous recombination at Alu repeat sequences. Proceedings of the National Academy of Sciences. 1989;86(12):4619–4623. 10.1073/pnas.86.12.4619 PMC287322 2734312

[33] Fennell BJ, Naughton JA, Dempsey E, Bell A. Cellular and molecular actions of dinitroaniline and phosphorothioamidate herbicides on *Plasmodium falciparum*: Tubulin as a specific antimalarial target. Molecular and Biochemical Parasitology. 2006;145(2):226–238. Available from: http://www.sciencedirect.com/science/article/pii/S0166685105003579. 10.1016/j.molbiopara.2005.08.020 16406111

[34] Makler MT, Hinrichs DJ. Measurement of the lactate dehydrogenase activity of *Plasmodium falciparum* as an assessment of parasitemia. The American Journal of Tropical Medicine and Hygiene. 1993;48(2):205–210. 8447524 10.4269/ajtmh.1993.48.205

[35] Makler MT, Ries J, Williams J, Bancroft J, Piper R, Gibbins B, et al Parasite lactate dehydrogenase as an assay for *Plasmodium falciparum* drug sensitivity. The American Journal of Tropical Medicine and Hygiene. 1993;48(6):739–741. 8333566 10.4269/ajtmh.1993.48.739

[36] Cunningham E, Drag M, Kafarski P, Bell A. Chemical target validation studies of aminopeptidase in malaria parasites using *α*-aminoalkylphosphonate and phosphonopeptide inhibitors. Antimicrobial Agents and Chemotherapy. 2008;52(9):3221–3228. 10.1128/AAC.01327-07 18458130 PMC2533478

[37] Staalsoe T, Giha HA, Dodoo D, Theander TG, Hviid L. Detection of antibodies to variant antigens on *Plasmodium falciparum*-infected erythrocytes by flow cytometry. Cytometry. 1999;35(4):329–336. 10.1002/(SICI)1097-0320(19990401)35:4<329::AID-CYTO5>3.3.CO;2-P 10213198

[38] Baum J, Maier AG, Good RT, Simpson KM, Cowman AF. Invasion by P. falciparum merozoites suggests a hierarchy of molecular interactions. PLoS pathogens. 2005;1(4):e37 10.1371/journal.ppat.0010037 16362075 PMC1315277

[39] Parthasarathi L, Devocelle M, Søndergaard C, Baran I, O'Dushlaine CT, Davey NE, et al Absolute net charge and the biological activity of oligopeptides. Journal of Chemical Information and Modeling. 2006;46(5):2183–2190. 10.1021/ci0600760 16995748

[40] Trottein F, Cowman AF. Molecular cloning and sequence of two novel P-type adenosinetriphosphatases from *Plasmodium falciparum* . European Journal of Biochemistry. 1995 1;227(1–2):214–225. 10.1111/j.1432-1033.1995.tb20379.x 7851389

[41] Tarazona-Santos E, Castilho L, Amaral DRT, Costa DC, Furlani NG, Zuccherato LW, et al Population genetics of GYPB and association study between GYPB*S/s polymorphism and susceptibility to *P. falciparum* infection in the Brazilian Amazon. PLoS One. 2011 1;6(1):e16123 10.1371/journal.pone.0016123 21283638 PMC3026040

