# Supplementary material for: Design and Evaluation of Antimalarial Peptides Derived from Prediction of Short Linear Motifs in Proteins Related to Erythrocyte Invasion
Source: PLoS One. 2015 Jun 3;10(6):e0127383. doi: 10.1371/journal.pone.0127383 (PMC4454681; doi:10.1371/journal.pone.0127383)
Supplement: S1 File — Peptide 1, Ac-SEQKTPFNINRSK-pal Putative transporter protein Q8II64 (PF11_0310) 609 residues in length. Predicted SLiM: QKTPF (residues 78–82) (Figure A). Peptide 2, pal-KKKLYLYFELFF-NH2, and Peptide 3, pal-KKKLYLYFE-NH2 Putative protein kinase Q8ILC4 (PF14_0320) 1518 residues in length. Predicted SLiM: YLYFE (residues 444–448) (Figure B). Peptide 4, pal-KRKLKEEQRTKKIKID Putative Calcium-transporting ATPase Q76NN8 (PFA0310c) 1228 residues in length. Predicted SLiM: KKIKI (residues 1223–1227) (Figure C). Peptide 5, pal-SSSRKNRFRYLPF-NH2 Putative aminophospholipid-transporting P-ATPase Q8I5L4 (PFL0950c) 1555 residues in length. Predicted SLiM: RFRYLP (residues 463–468) (Figure D). Peptide 6, pal-KNSNEPHHIFNIFQK-NH2 Reticulocyte binding protein homolog 4 C0H496. 1716 residues in length. Predicted SLiM: FNIFQ (residues 1659–1663) (Figure E). Peptide 7, pal-KEEIIEIVFDENEEKYF Reticulocyte binding protein 2 C0H5F4 (RBP2B_PLAF7) 3179 residues in length. Predicted SLiM: IEIVFDE (residues 3167–3173) (Figure F). Peptide 8, Ac-LSESIKNLLKNIYKK-NH2 Serine repeat antigen 4 O96164 (O96164_PLAF7) 962 residues in length. Predicted SLiM: LLKNIYKK (residues 328–335) (Figure G). Peptide 9, pal-YEKRRKPEDVL-NH2 Basigin P35613 (BASI_HUMAN) 385 residues in length (Figure H). Peptide 10, pal-RRLIKKSP-NH2 Glycophorin-A P02724 (GLPA_HUMAN) 150 residues in length (Figure I). Peptide 11, pal-SYSIRRLIKA and peptide 12, pal-SYTIRRLIKA Glycophorin-B P06028 (GLPB_HUMAN) 91 residues in length (Figure J). Peptide 13, pal-KHRKGNNA-NH2 Complement receptor type 1 P17927 (CR1_HUMAN) 2039 residues in length (Figure K). (PDF) [file pone.0127383.s001.pdf]

In Silico Design and Evaluation of Antimalarial Peptides Derived from Prediction of Short Linear Motifs in Proteins Related to Erythrocyte Invasion – Supporting Information

Alessandra Bianchin[1,2,3,4], Angus Bell[4], Anthony J. Chubb[1,2,3], Nathalie Doolan[3], Darren Leneghan[4], Denis C. Shields[1,2,3], Ilias Stavropoulos[1,2,3], Catherine Mooney[1,2,3,*]

1 Conway Institute of Biomolecular and Biomedical Science, University College Dublin, Dublin, Ireland

2 Complex and Adaptive Systems Laboratory, University College Dublin, Dublin, Ireland

3 School of Medicine and Medical Science, University College Dublin, Dublin, Ireland

4 Department of Microbiology, School of Genetics and Microbiology, Moyne Institute of Preventive Medicine, Trinity College, Dublin, Ireland

∗ **E-mail: catherine.mooney@ucd.ie**

Images from the SLiMPred server showing the predicted SLiMs and peptides.

The images below are taken from the SLiMPred server output and have been edited to show the portion of the protein sequence around the predicted SLiM. The protein sequence runs from left to right, N- to C-terminus. The section of the protein sequence shown is indicated in the top left hand corner of the plot. The height of the green bars is between 0 and 1, the higher the bar (i.e. the closer to 1), the more likely the residue is to be part of a SliM. In all cases, if there is a transmembrane domain, we have selected the peptide from what we believe to be the cytoplasmic portion of the protein. Table A lists the species found in the alignments produced by the SLiMPred server and Table B shows the colour coding of the amino acids in the alignments.

Code	Taxon Node	Official (scientific) name
BABBO	5865	Babesia bovis
CAEBR	6238	Caenorhabditis briggsae
CAERE	31234	Caenorhabditis remanei
CALJA	9483	Callithrix jacchus
CANFA	9615	Canis familiaris
CAVPO	10141	Cavia porcellus
CIOIN	7719	Ciona intestinalis
CRYPV	5807	Cryptosporidium parvum
DANRE	7955	Danio rerio
DIPOR	10020	Dipodomys ordii
EQUPR	9798	Equus przewalskii
GASAC	69293	Gasterosteus aculeatus
GORGO	9595	Gorilla gorilla gorilla
LOXAF	9785	Loxodonta africana
MACMU	9544	Macaca mulatta
MICMU	30608	Microcebus murinus
MONDO	13616	Monodelphis domestica
MOUSE	10090	Mus musculus
ORYLA	8090	Oryzias latipes
PANTR	9598	Pan troglodytes
PIG	9823	Sus scrofa
PLABA	5823	Plasmodium berghei (strain Anka)
PLACH	5825	Plasmodium chabaudi
PLAF7	36329	Plasmodium falciparum (isolate 3D7)
PLAKH	5851	Plasmodium knowlesi (strain H)
PLAVS	126793	Plasmodium vivax (strain Salvador I)
PLAYO	73239	Plasmodium yoelii yoelii
PONPY	9600	Pongo pygmaeus
RAT	10116	Rattus norvegicus
SPETR	43179	Spermophilus tridecemlineatus
TAEGU	59729	Taeniopygia guttata
TARSY	9478	Tarsius syrichta
TETNG	99883	Tetraodon nigroviridis
THEAN	5874	Theileria annulata
THEPA	5875	Theileria parva

Table A. List of species found in the alignments produced by the SLiMPred server.

Amino Acid			Colour	Property
Alanine	ALA	A	Blue	Hydrophobic
Phenylalanine	PHE	F	Blue	Hydrophobic
Isoleucine	ILE	I	Blue	Hydrophobic
Leucine	LEU	L	Blue	Hydrophobic
Methionine	MET	M	Blue	Hydrophobic
Valine	VAL	V	Blue	Hydrophobic
Tryptophan	TRP	W	Blue	Hydrophobic
Cysteine	CYS	C	Blue/pink	Hydrophobic/Hydrophilic
Histidine	HIS	H	Cyan	Aromatic
Tyrosine	TYR	Y	Cyan	Aromatic
Asparagine	ASN	N	Green	Neutral
Glutamine	GLN	Q	Green	Neutral
Serine	SER	S	Green	Neutral
Threonine	THR	T	Green	Neutral
Aspartic Acid	ASP	D	Magenta	Acidic
Glutamic Acid	GLU	E	Magenta	Acidic
Lysine	LYS	K	Red	Basic
Arginine	ARG	R	Red	Basic
Proline	PRO	P	Yellow	
Glycine	GLY	G	Brown	

Table B. Colour coding of the amino acids in the alignments.

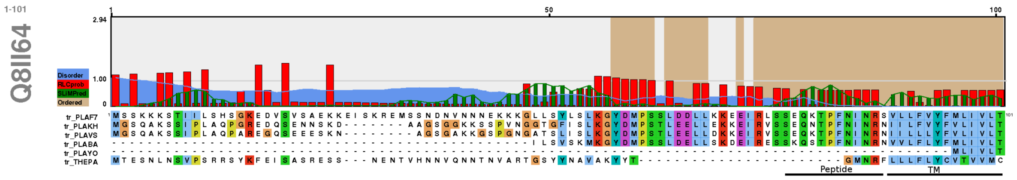

Figure A. Peptide 1: Ac-SEQKTPFNINRSK-pal Putative transporter protein Q8II64 (PF11_0310) 609 residues in length. Predicted SLiM: QKTPF (residues 78-82).

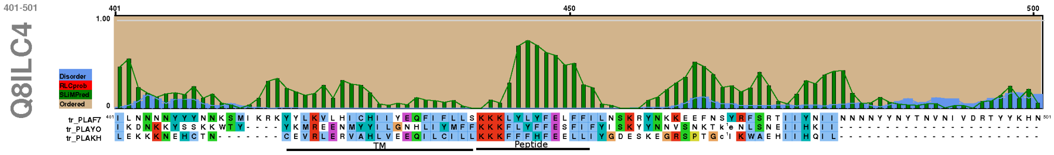

Figure B. Peptide 2, pal-KKKLYLYFELFF-NH$_2$, and Peptide 3, pal-KKKLYLYFE-NH$_2$ Putative protein kinase Q8ILC4 (PF14_0320) 1518 residues in length. Predicted SLiM: YLYFE (residues 444-448).

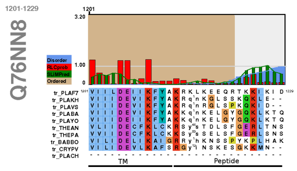

Figure C. Peptide 4, pal-KRKLKEEQRTKKIKID Putative Calcium-transporting ATPase Q76NN8 (PFA0310c) 1228 residues in length. Predicted SLiM: KKIKI (residues 1223-1227).

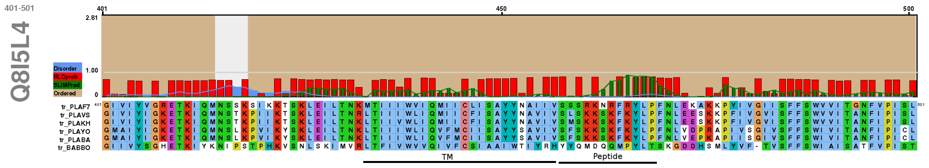

Figure D. Peptide 5, pal-SSSRKNRFRYLPF-NH$_2$ Putative aminophospholipid-transporting P-ATPase Q8I5L4 (PFL0950c) 1555 residues in length. Predicted SLiM: RFRYLP (residues 463-468).

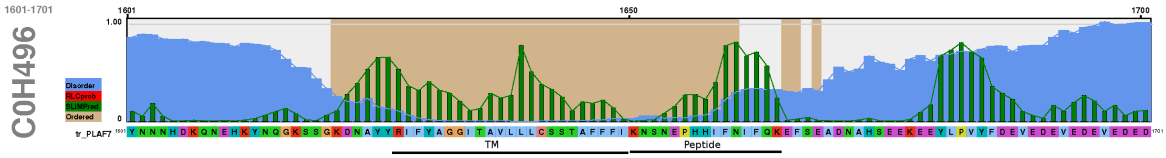

Figure E. Peptide 6, pal-KNSNEPHHIFNIFQK-NH$_2$ Reticulocyte binding protein homolog 4 C0H496. 1716 residues in length. Predicted SLiM: FNIFQ (residues 1659-1663).

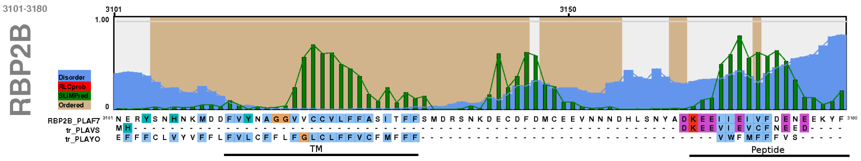

Figure F. Peptide 7, pal-KEEIIEIVFDENEEKYF Reticulocyte binding protein 2 C0H5F4 (RBP2B_PLAF7) 3179 residues in length. Predicted SLiM: IEIVFDE (residues 3167–3173).

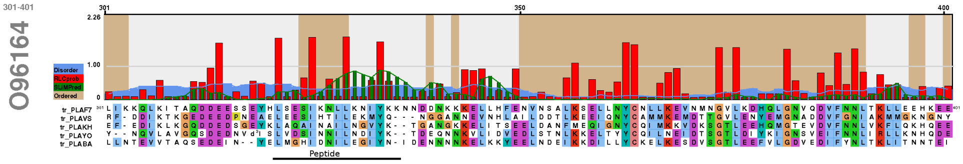

Figure G. Peptide 8, Ac-LSESIKNLLKNIYKK-NH$_2$ Serine repeat antigen 4 O96164 (O96164_PLAF7) 962 residues in length. Predicted SLiM: LLKNIYKK (residues 328–335).

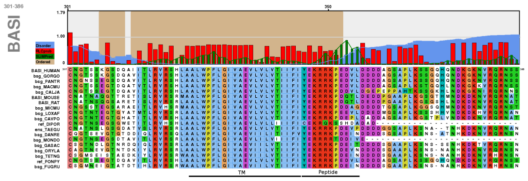

Figure H. Peptide 9, pal-YEKRRKPEDVL-NH$_2$ Basigin P35613 (BASI_HUMAN) 385 residues in length.

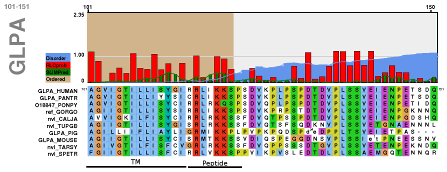

Figure I. Peptide 10, pal-RRLIKKSP-NH$_2$ Glycophorin-A P02724 (GLPA_HUMAN) 150 residues in length.

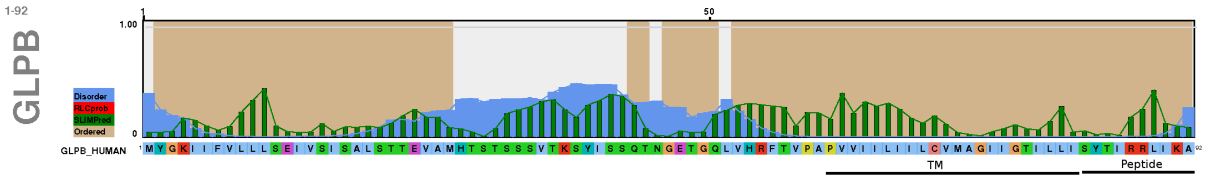

Figure J. Peptide 11, pal-SYSIRRLIKA and peptide 12, pal-SYTIRRLIKA Glycophorin-B P06028 (GLPB_HUMAN) 91 residues in length.

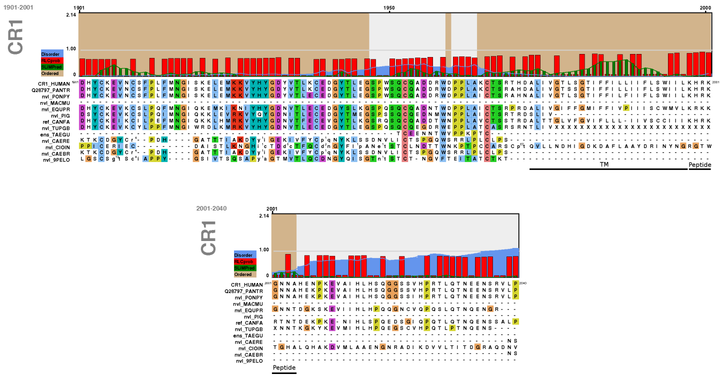

Figure K. Peptide 13, pal-KHRKGNNA-NH$_2$ Complement receptor type 1 P17927 (CR1_HUMAN) 2039 residues in length.
